# Cystal structure of *N*-[2-(benzo[*d*][1,3]dioxol-5-yl)eth­yl]-4-methyl­benzene­sulfonamide

**DOI:** 10.1107/S2056989015010555

**Published:** 2015-06-06

**Authors:** Ke-Bin Huang, Gui-Jie Zhang

**Affiliations:** a School of Chemistry and Chemical Engineering, Key Laboratory for the Chemistry and Molecular Engineering of Medicinal Resources (Ministry of Education), Guangxi Normal University, Guilin 541004, People’s Republic of China; b College of Pharmacy, Guilin Medical College, Guilin 541004, People’s Republic of China

**Keywords:** crystal structure, methyl­benzene­sulfonamide derivatives, hydrogen bonding

## Abstract

In the title compound, C_16_H_17_NO_4_S, the heterocyclic ring is almost planar (r.m.s. deviation = 0.007Å) and the dihedral angle between the benzene rings is 28.18 (10)°. The N—C—C—C torsion angle for the central chain is 62.4 (3)°: overall, the mol­ecule has a Z-shape. In the crystal, inversion dimers linked by pairs of N—H⋯O hydrogen bonds generate *R*_2_^2^(8) loops.

## Related literature   

For background to methyl­benzene­sulfonamide derivatives, see: Barn *et al.* (2001 ▸); Ghorai *et al.* (2010 ▸).
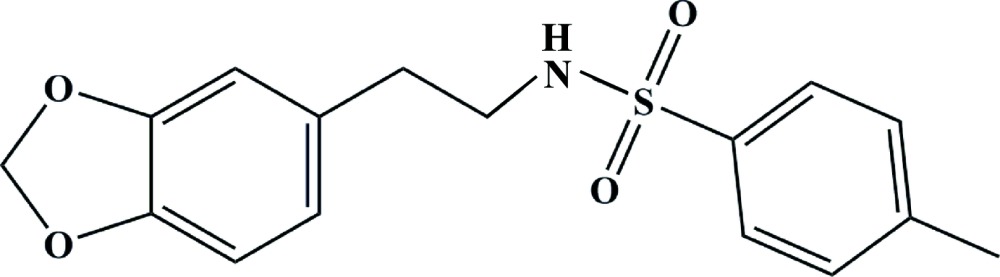


## Experimental   

### Crystal data  

C_16_H_17_NO_4_S
*M* *_r_* = 319.37
Monoclinic, 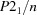

*a* = 12.3265 (2) Å
*b* = 9.96026 (16) Å
*c* = 12.7021 (3) Å
β = 100.5980 (18)°
*V* = 1532.90 (5) Å^3^
*Z* = 4
Mo *K*α radiation
μ = 0.23 mm^−1^
*T* = 293 K
0.40 × 0.20 × 0.12 mm

### Data collection  

Agilent SuperNova (single source at offset), Eos diffractometer
Absorption correction: multi-scan (*CrysAlis PRO*; Agilent, 2011 ▸) *T*_min_ = 0.868, *T*_max_ = 1.000
12219 measured reflections
3136 independent reflections
2587 reflections with *I* > 2σ(*I*)
*R*_int_ = 0.029

### Refinement  

*R*[*F* ^2^ > 2σ(*F* ^2^)] = 0.043
*wR*(*F* ^2^) = 0.115
*S* = 1.05
3136 reflections
204 parameters
H atoms treated by a mixture of independent and constrained refinement
Δρ_max_ = 0.19 e Å^−3^
Δρ_min_ = −0.39 e Å^−3^

### 

Data collection: *CrysAlis PRO* (Agilent, 2011 ▸); cell refinement: *CrysAlis PRO*; data reduction: *CrysAlis PRO*; program(s) used to solve structure: *SHELXS97* (Sheldrick, 2008 ▸); program(s) used to refine structure: *SHELXL97* (Sheldrick, 2008 ▸); molecular graphics: *OLEX* (Dolomanov *et al.*, 2009 ▸); software used to prepare material for publication: *OLEX*.

## Supplementary Material

Crystal structure: contains datablock(s) I, New_Global_Publ_Block. DOI: 10.1107/S2056989015010555/hb7436sup1.cif

Structure factors: contains datablock(s) I. DOI: 10.1107/S2056989015010555/hb7436Isup2.hkl

Supporting information file. DOI: 10.1107/S2056989015010555/hb7436Isup3.cml

**Fig. 1.**
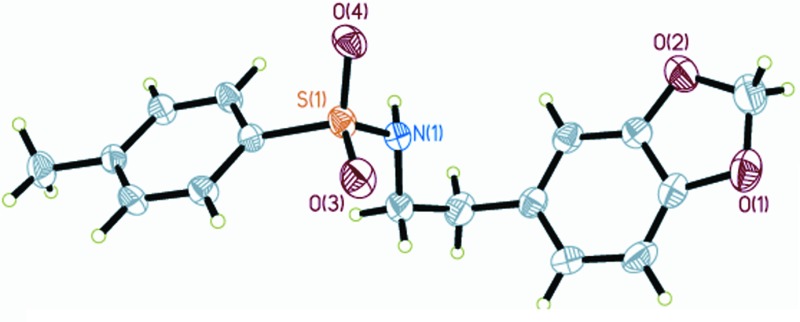
The molecular structure of title compound, with atom labelling. Displacement ellipsoids are drawn at 30% probability level.

. DOI: 10.1107/S2056989015010555/hb7436fig1.tif
The mol­ecular structure of title compound, with atom labelling. Displacement ellipsoids are drawn at 30% probability level.

CCDC reference: 1401539

Additional supporting information:  crystallographic information; 3D view; checkCIF report

## Figures and Tables

**Table 1:** Hydrogen-bond geometry (, )

*D*H*A*	*D*H	H*A*	*D* *A*	*D*H*A*
N1H1O4^i^	0.84(2)	2.19(2)	3.026(2)	172(2)

Symmetry code: (i) 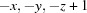.

## supplementary crystallographic information

### S1. Experimental

A solution of sulfonylchloride (10 mmol) in dichloromethane (15 mL) was slowly added to a cooled solution of methylenedioxyphenethylamine (15 mmol) in dichloromethane (10 ml) and triethylamine (15 mmol). Yellow blocks of the title compound were obtained by slow evaporation of a solution in methanol.

### S2. Refinement

Refinement of *F^2^* against ALL reflections. The weighted *R*-factor *wR* and goodness of fit S are based on F^2^, conventional *R*-factors *R* are based on F, with F set to zero for negative F^2^. The threshold expression of *F^2^* > 2sigma(*F^2^*) is used only for calculating *R*-factors(gt) *etc*. and is not relevant to the choice of reflections for refinement. *R*-factors based on *F^2^* are statistically about twice as large as those based on *F*, and *R*-factors based on ALL data will be even larger.

### Crystal data

**Table:**

C_16_H_17_NO_4_S	*F*(000) = 672
*M**_r_* = 319.37	*D*_x_ = 1.384 Mg m^−^^3^
Monoclinic, *P*2_1_/*n*	Mo *K*α radiation, λ = 0.7107 Å
*a* = 12.3265 (2) Å	Cell parameters from 5283 reflections
*b* = 9.96026 (16) Å	θ = 2.9–28.7°
*c* = 12.7021 (3) Å	µ = 0.23 mm^−^^1^
β = 100.5980 (18)°	*T* = 293 K
*V* = 1532.90 (5) Å^3^	Block, yellow
*Z* = 4	0.40 × 0.20 × 0.12 mm

### Data collection

**Table:**

Agilent SuperNova (single source at offset), Eos diffractometer	3136 independent reflections
Radiation source: SuperNova (Mo) X-ray Source	2587 reflections with *I* > 2σ(*I*)
Mirror monochromator	*R*_int_ = 0.029
Detector resolution: 16.1623 pixels mm^-1^	θ_max_ = 26.4°, θ_min_ = 2.9°
ω scans	*h* = −15→15
Absorption correction: multi-scan (*CrysAlis PRO*; Agilent, 2011)	*k* = −12→12
*T*_min_ = 0.868, *T*_max_ = 1.000	*l* = −15→15
12219 measured reflections	

### Refinement

**Table:**

Refinement on *F*^2^	Primary atom site location: structure-invariant direct methods
Least-squares matrix: full	Secondary atom site location: difference Fourier map
*R*[*F*^2^ > 2σ(*F*^2^)] = 0.043	Hydrogen site location: inferred from neighbouring sites
*wR*(*F*^2^) = 0.115	H atoms treated by a mixture of independent and constrained refinement
*S* = 1.05	*w* = 1/[σ^2^(*F*_o_^2^) + (0.0499*P*)^2^ + 0.4898*P*] where *P* = (*F*_o_^2^ + 2*F*_c_^2^)/3
3136 reflections	(Δ/σ)_max_ < 0.001
204 parameters	Δρ_max_ = 0.19 e Å^−^^3^
0 restraints	Δρ_min_ = −0.39 e Å^−^^3^

### Special details

**Table:**

Geometry. All e.s.d.'s (except the e.s.d. in the dihedral angle between two l.s. planes) are estimated using the full covariance matrix. The cell e.s.d.'s are taken into account individually in the estimation of e.s.d.'s in distances, angles and torsion angles; correlations between e.s.d.'s in cell parameters are only used when they are defined by crystal symmetry. An approximate (isotropic) treatment of cell e.s.d.'s is used for estimating e.s.d.'s involving l.s. planes.
Refinement. Refinement of *F*^2^ against ALL reflections. The weighted *R*-factor *wR* and goodness of fit *S* are based on *F*^2^, conventional *R*-factors *R* are based on *F*, with *F* set to zero for negative *F*^2^. The threshold expression of *F*^2^ > σ(*F*^2^) is used only for calculating *R*-factors(gt) *etc*. and is not relevant to the choice of reflections for refinement. *R*-factors based on *F*^2^ are statistically about twice as large as those based on *F*, and *R*- factors based on ALL data will be even larger.

### Fractional atomic coordinates and isotropic or equivalent isotropic displacement parameters (Å^2^)

**Table:**

	*x*	*y*	*z*	*U*_iso_*/*U*_eq_	
S1	0.01040 (4)	0.17100 (5)	0.62290 (4)	0.04836 (17)	
O1	0.10241 (14)	−0.39697 (19)	0.99233 (14)	0.0834 (5)	
O2	0.07337 (17)	−0.43875 (18)	0.81134 (15)	0.0899 (6)	
O3	0.04945 (13)	0.23231 (16)	0.72422 (12)	0.0689 (4)	
O4	0.08943 (10)	0.12122 (14)	0.56207 (12)	0.0602 (4)	
N1	−0.06387 (14)	0.04327 (17)	0.64128 (15)	0.0521 (4)	
H1	−0.0777 (17)	−0.003 (2)	0.5849 (18)	0.058 (7)*	
C1	0.1333 (2)	−0.4786 (3)	0.9109 (2)	0.0844 (8)	
H1A	0.2117	−0.4692	0.9115	0.101*	
H1B	0.1181	−0.5721	0.9239	0.101*	
C2	0.02598 (17)	−0.3080 (2)	0.93953 (17)	0.0564 (5)	
C3	−0.0287 (2)	−0.2082 (3)	0.98013 (18)	0.0725 (7)	
H3	−0.0176	−0.1910	1.0533	0.087*	
C4	−0.10220 (19)	−0.1330 (2)	0.90745 (18)	0.0657 (6)	
H4	−0.1404	−0.0633	0.9331	0.079*	
C5	−0.12075 (16)	−0.1574 (2)	0.79891 (16)	0.0515 (5)	
C6	−0.06264 (17)	−0.2603 (2)	0.76031 (16)	0.0556 (5)	
H6	−0.0725	−0.2786	0.6874	0.067*	
C7	0.00883 (17)	−0.3334 (2)	0.83196 (17)	0.0538 (5)	
C8	−0.20189 (18)	−0.0740 (2)	0.7228 (2)	0.0676 (6)	
H8A	−0.2671	−0.0597	0.7541	0.081*	
H8B	−0.2244	−0.1242	0.6569	0.081*	
C9	−0.15798 (18)	0.0611 (2)	0.6958 (2)	0.0654 (6)	
H9A	−0.2159	0.1108	0.6499	0.078*	
H9B	−0.1351	0.1124	0.7610	0.078*	
C10	−0.07539 (13)	0.28694 (17)	0.54277 (14)	0.0397 (4)	
C11	−0.08585 (15)	0.41631 (18)	0.57810 (15)	0.0462 (4)	
H11	−0.0486	0.4434	0.6451	0.055*	
C12	−0.15261 (15)	0.50503 (18)	0.51218 (16)	0.0491 (4)	
H12	−0.1592	0.5927	0.5354	0.059*	
C13	−0.20962 (14)	0.46749 (17)	0.41312 (15)	0.0437 (4)	
C14	−0.28241 (19)	0.5655 (2)	0.34252 (19)	0.0665 (6)	
H14A	−0.2651	0.5636	0.2719	0.100*	
H14B	−0.2702	0.6543	0.3717	0.100*	
H14C	−0.3583	0.5411	0.3389	0.100*	
C15	−0.19811 (16)	0.33719 (18)	0.37970 (15)	0.0505 (5)	
H15	−0.2365	0.3099	0.3132	0.061*	
C16	−0.13115 (16)	0.24717 (18)	0.44277 (15)	0.0490 (5)	
H16	−0.1233	0.1602	0.4186	0.059*	

### Atomic displacement parameters (Å^2^)

**Table:**

	*U*^11^	*U*^22^	*U*^33^	*U*^12^	*U*^13^	*U*^23^
S1	0.0424 (3)	0.0496 (3)	0.0495 (3)	0.00838 (19)	−0.0009 (2)	0.0022 (2)
O1	0.0771 (11)	0.0969 (13)	0.0694 (11)	0.0077 (10)	−0.0045 (9)	0.0205 (10)
O2	0.1130 (14)	0.0765 (11)	0.0788 (12)	0.0330 (10)	0.0134 (11)	−0.0013 (10)
O3	0.0695 (9)	0.0724 (10)	0.0540 (9)	0.0114 (8)	−0.0171 (7)	−0.0045 (8)
O4	0.0426 (7)	0.0613 (8)	0.0773 (10)	0.0105 (6)	0.0124 (7)	0.0019 (7)
N1	0.0576 (10)	0.0477 (9)	0.0524 (10)	0.0109 (7)	0.0135 (8)	0.0075 (8)
C1	0.0721 (16)	0.0828 (18)	0.100 (2)	0.0123 (14)	0.0206 (15)	0.0238 (16)
C2	0.0544 (11)	0.0642 (13)	0.0483 (11)	−0.0089 (10)	0.0031 (9)	0.0088 (10)
C3	0.0907 (18)	0.0882 (17)	0.0389 (11)	−0.0001 (14)	0.0124 (11)	−0.0021 (12)
C4	0.0749 (14)	0.0733 (14)	0.0537 (13)	0.0090 (12)	0.0244 (11)	0.0001 (11)
C5	0.0477 (10)	0.0564 (11)	0.0514 (11)	−0.0090 (8)	0.0115 (9)	0.0062 (9)
C6	0.0674 (13)	0.0554 (12)	0.0416 (11)	−0.0091 (10)	0.0037 (9)	−0.0052 (9)
C7	0.0590 (12)	0.0484 (11)	0.0537 (12)	−0.0069 (9)	0.0100 (9)	−0.0037 (9)
C8	0.0518 (12)	0.0843 (16)	0.0671 (14)	0.0040 (11)	0.0118 (10)	0.0182 (12)
C9	0.0657 (13)	0.0674 (14)	0.0676 (14)	0.0216 (11)	0.0244 (11)	0.0168 (11)
C10	0.0367 (8)	0.0398 (9)	0.0414 (9)	0.0008 (7)	0.0039 (7)	0.0008 (7)
C11	0.0465 (10)	0.0457 (10)	0.0441 (10)	−0.0004 (8)	0.0020 (8)	−0.0077 (8)
C12	0.0532 (10)	0.0364 (9)	0.0562 (12)	0.0027 (8)	0.0059 (9)	−0.0071 (8)
C13	0.0414 (9)	0.0421 (9)	0.0475 (10)	0.0016 (7)	0.0079 (8)	0.0052 (8)
C14	0.0713 (14)	0.0559 (12)	0.0669 (14)	0.0134 (10)	−0.0019 (11)	0.0107 (11)
C15	0.0574 (11)	0.0472 (10)	0.0417 (10)	0.0022 (8)	−0.0043 (8)	−0.0042 (8)
C16	0.0574 (11)	0.0382 (9)	0.0476 (11)	0.0040 (8)	−0.0005 (9)	−0.0057 (8)

### Geometric parameters (Å, º)

**Table:**

S1—O3	1.4259 (15)	C6—C7	1.356 (3)
S1—O4	1.4383 (14)	C8—H8A	0.9700
S1—N1	1.6092 (18)	C8—H8B	0.9700
S1—C10	1.7584 (17)	C8—C9	1.513 (3)
O1—C1	1.421 (3)	C9—H9A	0.9700
O1—C2	1.375 (3)	C9—H9B	0.9700
O2—C1	1.400 (3)	C10—C11	1.378 (2)
O2—C7	1.371 (3)	C10—C16	1.386 (2)
N1—H1	0.84 (2)	C11—H11	0.9300
N1—C9	1.467 (3)	C11—C12	1.380 (3)
C1—H1A	0.9700	C12—H12	0.9300
C1—H1B	0.9700	C12—C13	1.375 (3)
C2—C3	1.355 (3)	C13—C14	1.505 (3)
C2—C7	1.367 (3)	C13—C15	1.381 (3)
C3—H3	0.9300	C14—H14A	0.9600
C3—C4	1.388 (3)	C14—H14B	0.9600
C4—H4	0.9300	C14—H14C	0.9600
C4—C5	1.377 (3)	C15—H15	0.9300
C5—C6	1.390 (3)	C15—C16	1.372 (3)
C5—C8	1.507 (3)	C16—H16	0.9300
C6—H6	0.9300		
			
O3—S1—O4	118.88 (9)	C5—C8—H8B	108.7
O3—S1—N1	108.32 (10)	C5—C8—C9	114.39 (18)
O3—S1—C10	107.96 (9)	H8A—C8—H8B	107.6
O4—S1—N1	105.37 (9)	C9—C8—H8A	108.7
O4—S1—C10	108.07 (9)	C9—C8—H8B	108.7
N1—S1—C10	107.79 (8)	N1—C9—C8	110.25 (17)
C2—O1—C1	105.37 (18)	N1—C9—H9A	109.6
C7—O2—C1	105.8 (2)	N1—C9—H9B	109.6
S1—N1—H1	109.7 (15)	C8—C9—H9A	109.6
C9—N1—S1	119.55 (15)	C8—C9—H9B	109.6
C9—N1—H1	114.4 (15)	H9A—C9—H9B	108.1
O1—C1—H1A	109.8	C11—C10—S1	120.52 (13)
O1—C1—H1B	109.8	C11—C10—C16	120.34 (16)
O2—C1—O1	109.2 (2)	C16—C10—S1	119.13 (13)
O2—C1—H1A	109.8	C10—C11—H11	120.6
O2—C1—H1B	109.8	C10—C11—C12	118.82 (17)
H1A—C1—H1B	108.3	C12—C11—H11	120.6
C3—C2—O1	129.2 (2)	C11—C12—H12	119.1
C3—C2—C7	121.3 (2)	C13—C12—C11	121.88 (17)
C7—C2—O1	109.5 (2)	C13—C12—H12	119.1
C2—C3—H3	121.5	C12—C13—C14	121.13 (17)
C2—C3—C4	116.9 (2)	C12—C13—C15	118.21 (16)
C4—C3—H3	121.5	C15—C13—C14	120.67 (18)
C3—C4—H4	118.7	C13—C14—H14A	109.5
C5—C4—C3	122.5 (2)	C13—C14—H14B	109.5
C5—C4—H4	118.7	C13—C14—H14C	109.5
C4—C5—C6	118.8 (2)	H14A—C14—H14B	109.5
C4—C5—C8	120.9 (2)	H14A—C14—H14C	109.5
C6—C5—C8	120.3 (2)	H14B—C14—H14C	109.5
C5—C6—H6	120.9	C13—C15—H15	119.4
C7—C6—C5	118.18 (19)	C16—C15—C13	121.29 (17)
C7—C6—H6	120.9	C16—C15—H15	119.4
C2—C7—O2	110.14 (19)	C10—C16—H16	120.3
C6—C7—O2	127.6 (2)	C15—C16—C10	119.46 (16)
C6—C7—C2	122.2 (2)	C15—C16—H16	120.3
C5—C8—H8A	108.7		
			
S1—N1—C9—C8	−169.30 (16)	C3—C2—C7—C6	0.5 (3)
S1—C10—C11—C12	178.98 (14)	C3—C4—C5—C6	−0.9 (3)
S1—C10—C16—C15	−179.88 (15)	C3—C4—C5—C8	179.5 (2)
O1—C2—C3—C4	−179.9 (2)	C4—C5—C6—C7	0.9 (3)
O1—C2—C7—O2	−0.2 (2)	C4—C5—C8—C9	79.5 (3)
O1—C2—C7—C6	−179.87 (19)	C5—C6—C7—O2	179.6 (2)
O3—S1—N1—C9	55.48 (18)	C5—C6—C7—C2	−0.8 (3)
O3—S1—C10—C11	6.21 (18)	C5—C8—C9—N1	62.4 (3)
O3—S1—C10—C16	−174.78 (15)	C6—C5—C8—C9	−100.1 (2)
O4—S1—N1—C9	−176.30 (16)	C7—O2—C1—O1	1.0 (3)
O4—S1—C10—C11	−123.57 (16)	C7—C2—C3—C4	−0.4 (3)
O4—S1—C10—C16	55.44 (17)	C8—C5—C6—C7	−179.48 (18)
N1—S1—C10—C11	123.02 (16)	C10—S1—N1—C9	−61.09 (18)
N1—S1—C10—C16	−57.97 (17)	C10—C11—C12—C13	0.7 (3)
C1—O1—C2—C3	−179.6 (2)	C11—C10—C16—C15	−0.9 (3)
C1—O1—C2—C7	0.8 (2)	C11—C12—C13—C14	179.75 (19)
C1—O2—C7—C2	−0.5 (3)	C11—C12—C13—C15	−0.5 (3)
C1—O2—C7—C6	179.1 (2)	C12—C13—C15—C16	−0.5 (3)
C2—O1—C1—O2	−1.1 (3)	C13—C15—C16—C10	1.1 (3)
C2—C3—C4—C5	0.6 (4)	C14—C13—C15—C16	179.33 (19)
C3—C2—C7—O2	−179.8 (2)	C16—C10—C11—C12	0.0 (3)

### Hydrogen-bond geometry (Å, º)

**Table:**

*D*—H···*A*	*D*—H	H···*A*	*D*···*A*	*D*—H···*A*
N1—H1···O4^i^	0.84 (2)	2.19 (2)	3.026 (2)	172 (2)

Symmetry code: (i) −*x*, −*y*, −*z*+1.
