# Supplementary figures and images for: Cystal structure of N-[2-(benzo[d][1,3]dioxol-5-yl)eth­yl]-4-methyl­benzene­sulfonamide
Source: Acta Crystallogr E Crystallogr Commun. 2015 Jun 6;71(Pt 7):o452. doi: 10.1107/S2056989015010555 (PMC4518912; doi:10.1107/S2056989015010555)

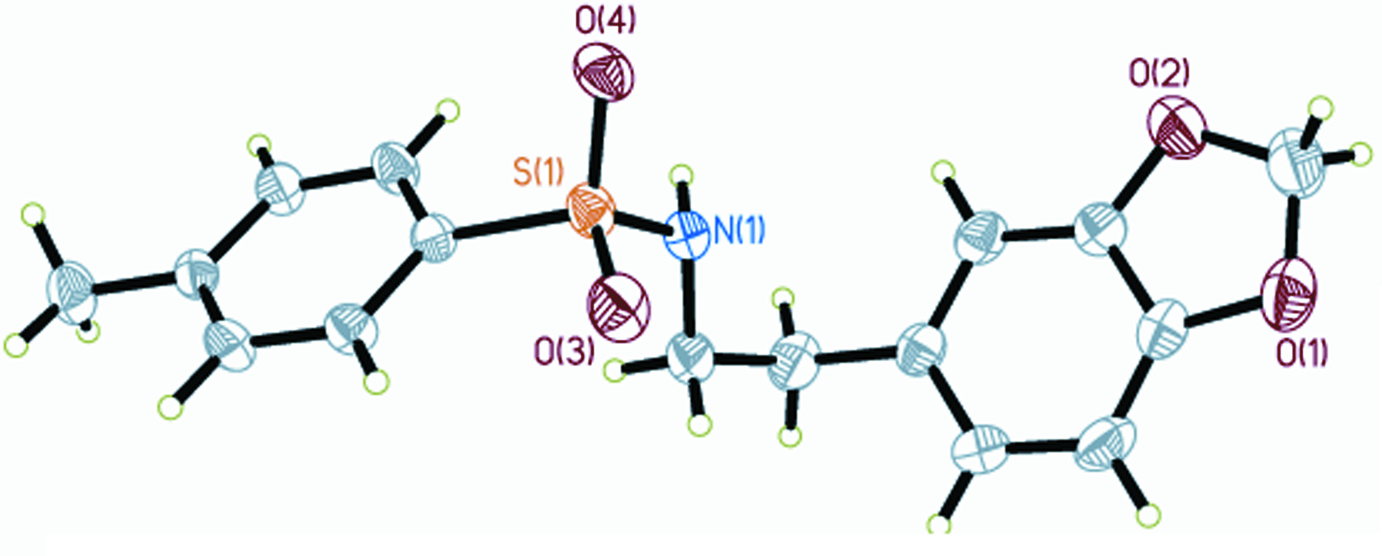

Supplement: Supplementary file 4 [file e-71-0o452-fig1.tif]
